# Enhancers in the *Peril* lincRNA locus regulate distant but not local genes

**DOI:** 10.1186/s13059-018-1589-8

**Published:** 2018-12-11

**Authors:** Abigail F. Groff, A. Rasim Barutcu, Jordan P. Lewandowski, John L. Rinn

**Affiliations:** 1 Department of Stem Cell and Regenerative Biology, Harvard University, Cambridge, MA 02138 USA; 2 Department of Systems Biology, Harvard Medical School, Boston, MA 02115 USA; 3 Donnelly Centre, University of Toronto, Toronto, ON M5S 3E1 Canada; 4 Department of Biochemistry, BioFrontiers, University of Colorado Boulder, Boulder, CO 80301 USA

**Keywords:** lincRNA, Mouse models, MPRA, *Cis*-regulation, Enhancer

## Abstract

**Background:**

Recently, it has become clear that some promoters function as long-range regulators of gene expression. However, direct and quantitative assessment of enhancer activity at long intergenic noncoding RNA (lincRNA) or mRNA gene bodies has not been performed. To unbiasedly assess the enhancer capacity across lincRNA and mRNA loci, we performed a massively parallel reporter assay (MPRA) on six lincRNA loci and their closest protein-coding neighbors.

**Results:**

For both gene classes, we find significantly more MPRA activity in promoter regions than in gene bodies. However, three lincRNA loci, *Lincp21*, *LincEnc1*, and *Peril*, and one mRNA locus, *Morc2a*, display significant enhancer activity within their gene bodies. We hypothesize that such peaks may mark long-range enhancers, and test this in vivo using RNA sequencing from a knockout mouse model and high-throughput chromosome conformation capture (Hi-C). We find that ablation of a high-activity MPRA peak in the *Peril* gene body leads to consistent dysregulation of *Mccc1* and *Exosc9* in the neighboring topologically associated domain (TAD). This occurs irrespective of *Peril* lincRNA expression, demonstrating this regulation is DNA-dependent. Hi-C confirms long-range contacts with the neighboring TAD, and these interactions are altered upon *Peril* knockout. Surprisingly, we do not observe consistent regulation of genes within the local TAD. Together, these data suggest a long-range enhancer-like function for the *Peril* gene body.

**Conclusions:**

A multi-faceted approach combining high-throughput enhancer discovery with genetic models can connect enhancers to their gene targets and provides evidence of inter-TAD gene regulation.

**Electronic supplementary material:**

The online version of this article (10.1186/s13059-018-1589-8) contains supplementary material, which is available to authorized users.

## Background

While a number of lincRNAs function by RNA-based mechanisms [1], recently, it has become clear that lincRNA loci also harbor functional DNA regulatory elements [2–7]. These DNA regions may account for the majority of the regulatory capacity of a lincRNA locus [3, 4]. To date, most efforts to characterize DNA elements within lincRNA loci have only focused on promoter regions [8–12] or predictions from genome-wide binding of p300 or H3K4me1 and H3K4me3 marks [13, 14]. The former approach has highlighted the crosstalk between neighboring lincRNA and mRNA promoters, suggesting that promoters of both mRNAs and lincRNAs can function as long-range enhancers to regulate transcription [15]. Similarly, mRNA loci have been shown to contain DNA enhancers in introns, UTRs, and even exons [12, 13, 16–20]. These studies unearth a complex regulatory landscape in both mRNA and lincRNA loci that requires multiple experimental and analytical approaches to properly decouple the contributions of DNA, transcription, RNA, and protein to regulation of gene expression. However, despite the success of these studies, our understanding of the contributions of potential enhancer elements lying within gene bodies to this complex regulatory landscape remains incomplete.

To disentangle the contributions of RNA and DNA at a set of developmentally regulated lincRNA loci, we previously generated 18 lincRNA knockout mouse models [21, 57]. In these models, the gene body is replaced by a *LacZ* reporter that is downstream of the endogenous promoter [21]. Thus, regulation of neighboring genes (*cis-*like regulation) in tissues where the reporter is not expressed implicates DNA and not the act of transcription or RNA product as the regulatory driver. This is because when a region is transcriptionally silent, neither transcription nor an RNA product is present to influence gene expression. Thus, such models allow for the direct detection of DNA-based regulatory elements.

Using these mouse models, we identified several lincRNA loci, including *Crnde*, *LincEnc1*, *Lincp21*, and *Tug1*, which appear to function as *cis*-regulators controlling expression in their genomic neighborhoods [6, 22]. However, it remains unclear if these regulatory roles are due to RNA-mediated interactions or underlying DNA. To directly interrogate these loci for DNA-based enhancer activity, we performed a massively parallel reporter assay (MPRA) using oligonucleotides that tile the promoters and gene bodies of six lincRNA and mRNA gene pairs. These loci include the four potential *cis*-acting lincRNA loci (*Crnde*, *LincEnc1*, *Lincp21*, and *Tug1*) and two lincRNA loci with undetermined regulatory function (*Peril* and *Fendrr*), as well as their closest protein-coding neighbors [6, 21]. Using this method, we show that the majority of MPRA-based enhancer activity in these loci arises from the promoter regions (mRNAs and lincRNAs alike). However, we also identify four loci, *Morc2a* (mRNA), *Lincp21, LincEnc1*, and *Peril* (lincRNAs), which harbor high MPRA activity in their gene bodies, consistent with promoter-independent enhancer activity.

To determine whether gene body MPRA peaks indicate enhancer activity in vivo, we used a previously generated *Peril* knockout mouse model to dissect the DNA regulatory roles of the gene body, excluding the promoter, at this locus. We focused on *Peril* because it has one of the highest gene body MPRA peaks and because it overlaps with a super-enhancer for *Sox2*, which is a critical stem cell regulator [23–25]. Surprisingly, upon deletion of *Peril*, we do not observe significant dysregulation of nearby genes (e.g., *Sox2*). Instead, we find that two genes, *Exosc9* and *Mccc1*, which are distally located (~ 1.5 Mb away) from the *Peril* locus in the neighboring TAD, are significantly downregulated in all four tissues examined. The downregulation of these genes occurred whether the *Peril* region was transcribed or silent. Moreover, comparison of high-throughput chromosome capture (Hi-C) data from wild-type and *Peril* knockout murine embryonic stem cells (mESCs) revealed alterations in physical long-range interactions between the *Peril* region and the TAD containing *Mccc1* and *Exosc9* genes. Taken together, our results quantify the presence of gene body DNA regulatory elements within lincRNA loci and identify their corresponding candidate target genes.

## Results

### MPRA to interrogate locus activity

**Fig. 1:**
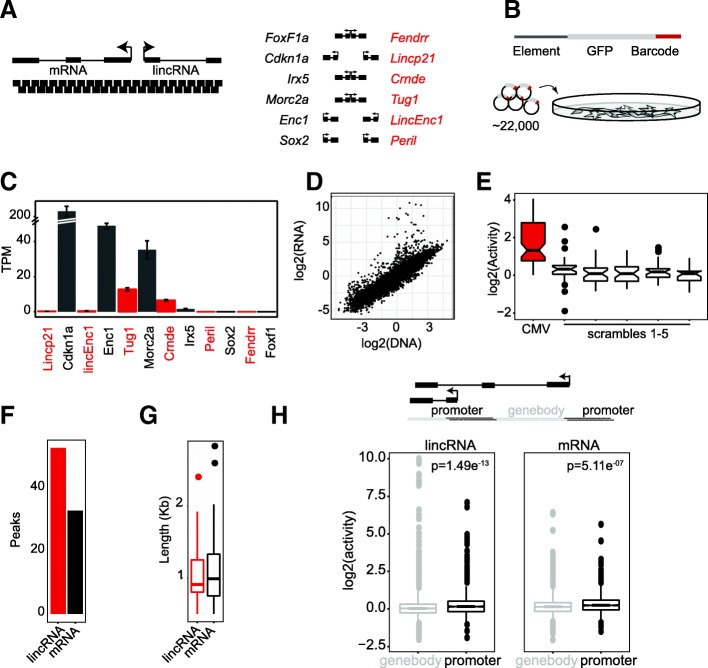
MPRA in C2C12 cells identifies sequences with differential enhancer activity. **a** Summary of MPRA pool design. *Cis-*acting lincRNAs (right) and their closest protein-coding neighbor (left) are redundantly tiled by 90-bp windows starting every 50 bp. **b** Core oligo design and experimental overview. Actual oligos flanked by universal primer sites for amplification. Element corresponds to 90-bp genomic sequence, barcode is a 10nt unique identifying tag, and GFP with minimal promoter is inserted after restriction enzyme digestion. **c** C2C12 expression (TPM) for each gene in the MPRA pool. Red indicates lincRNA, and gray indicates mRNA. **d** Median sample scatter plot of C2C12 RNA to DNA input control barcode counts (median across replicates used for each barcode, normalized for sequencing depth). **e** Boxplots of relative signal originating from CMV-promoter-tiling barcodes or oligos tiling five different scrambled versions of the same sequence. *Y*-axis shows log_2_(activity), i.e., log_2_(normed RNA counts/normed DNA counts). **f** Total number of significant regions identified for lincRNAs (red) or mRNAs (gray). **g** Size distribution of significant regions identified for lincRNAs (red) or mRNAs (gray). **h** Boxplot of gene body- or promoter-originating oligos from lincRNA loci. The gene body is represented in gray, and the promoter is represented in black. Right, same for mRNA loci. Top illustration: Scheme of TSS-based oligo partitioning. All oligos 1000 bp upstream of a TSS are labeled “promoter” (in black), and the remaining oligos are considered “gene body” (in gray)

To determine whether lincRNA loci might contain enhancer activity in their gene bodies, we performed an MPRA screen of six lincRNAs: *Crnde*, *Fendrr*, *LincEnc1*, *Lincp21*, *Peril*, and *Tug1.* To directly compare lincRNA with mRNA loci, we also included the nearest protein-coding gene to each respective lincRNA: *Irx5*, *Foxf1a*, *Enc1*, *Cdkn1a*, *Sox2*, and *Morc2a*, respectively (Fig. 1a). This approach allowed us to precisely interrogate the enhancer capacity of the DNA sequence, offering a quantitative advantage over ChIP-based methods which are unable to measure the transcriptional activating potential of DNA.

The MPRA method relies on the synthesis of thousands of oligos spanning a genomic region of interest, and can be used to systematically dissect the location of regulatory elements [3, 26–28]. We generated ~ 22,000 distinct oligos tiling the 12 loci and their promoters (defined as 1 kb upstream of each transcriptional start site (TSS)) (Fig. 1a, Additional files 1 and 2). Each oligo is comprised of 90 nucleotides (nt) of genomic sequence, two restriction enzyme sites, a unique 10nt barcode sequence, and flanking universal primer sites. The oligo pool is then cloned into a minimal vector, and a GFP reporter is inserted. This results in a barcode-tagged GFP that is transcribed in transiently transfected cells and can be used as a readout of activity (Fig. 1b). To ensure high redundancy in our dataset, we tiled each locus in 50nt intervals and have designed the pool such that every 90nt genomic element is represented by five unique barcodes.

### MPRA activity is enriched in promoters

We performed five replicate transfections in C2C12 cells, a mouse myoblast cell line in which to the genes of interest have varied expression status (Fig. 1c). As a quality control measure, we assessed replicate correlation between RNA samples and between DNA control libraries (Additional file 3: Figure S1). Normalized replicate samples correlated well with one another (*R*^2^ > 0.99, Additional file 3: Figure S1), while exhibiting clear signal with respect to DNA input control (*R*^2^ ~ 0.8544, Fig. 1d). To evaluate the dynamic range of the MPRA, we included tiles across a promoter known to be highly active in this cell type (cytomegalovirus) and scrambled copies of this promoter. Tiles corresponding to the scrambled promoter exhibited significantly less activity than those corresponding to the proper sequence, indicating the specificity of the assay (Fig. 1e). Together, these results highlight the quality and reproducibility of our MPRA data.

In order to define significantly active MPRA regions, we leveraged our tiling design using a sliding window statistic (see the “Methods” section). Significant regions were calculated using a bootstrapped *p* value (BH-corrected *p* < 0.01, FDR 1%). Using this threshold, we found 45 unique peaks in our lincRNA loci and 28 peaks in our mRNA loci, highlighting that both types of loci can contain enhancers (Fig. 1f). Average peak size for lincRNA loci and mRNA loci was 1046 bp and 1127 bp, respectively (Fig. 1g).

Since many of the active MPRA regions occur near TSSs, we sought to compare the activity distribution of oligos in promoter regions with those in the gene bodies. To do this, we segmented the data by proximity to TSSs. Each oligo was labeled as either promoter-specific (within 1 kb upstream of a TSS) or gene body-specific (remaining oligos, Fig. 1h). We found that for both lincRNAs and mRNAs, oligos in promoter regions generated significantly more activity than those in gene bodies (*p* < 1e−6, Kolmogorov-Smirnov test, Fig. 1h). This finding is consistent with our peak-based observation, with the known functional modalities of classical promoters, and with the role of promoters in long distance gene regulation [10, 15]. Taken together, our results demonstrate that elements within lincRNA and mRNA promoters alike contribute to *cis*-regulatory activity more so than their gene body counterparts.

### MPRA activity in gene bodies identifies candidate enhancer loci

**Fig. 2:**
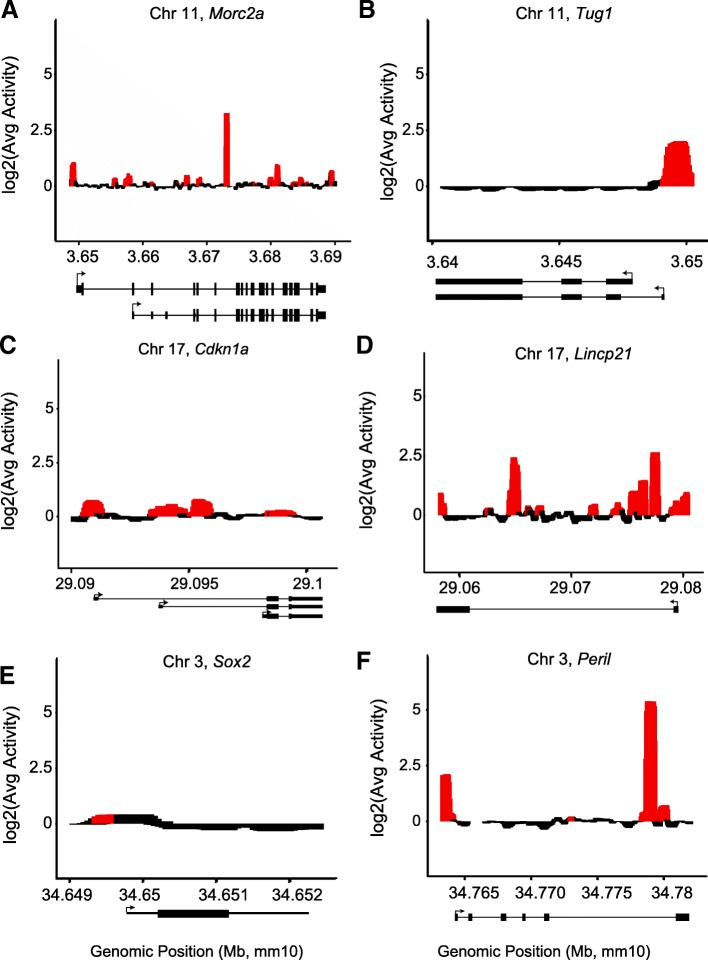
MPRA reveals enhancer activity and motifs in lincRNA loci. Smoothed signal plot for **a** protein-coding locus *Morc2a* and neighboring lincRNA *Tug1* (**b**). Red indicates significantly activated regions calculated with a window size of 500 bp and slide of 50 bp (see the “Methods” section). Genomic position (in Mb, mm10) across the *X*-axis, with gene structures indicated below. *Y*-axis represents log_2_(activity). **c**–**f** Same as **a** and **b** for the *Cdkn1a*, *Lincp21*, *Sox2*, and *Peril* loci

While the majority of activity lies in promoter regions, 9 of 12 loci showed significant gene body activity (Fig. 2, Additional file 3: Figure S2). We highlight six loci, three lincRNAs and three mRNAs, demonstrating a range of MPRA activity profiles (Fig. 2). The protein-coding gene *Morc2a* contains 16 peaks (Fig. 2a), a finding consistent with the literature on intronic enhancers in mRNA loci [13, 16–20]. The lincRNA *Tug1* shares a promoter with *Morc2a*, but has a dramatically different enhancer profile. While its promoter is active, there are no peaks of activity across its gene body (Fig. 2b). The protein-coding gene *Cdkn1a* contains four regions of enhancer activity (Fig. 2c), two of which are near TSSs. The neighboring *Lincp21* locus also contains enhancer activity in both its promoter and gene body (Fig. 2d). The protein-coding gene *Sox2*, which is not expressed in C2C12 cells, has minimal activity in its promoter and no activity across its gene body (Fig. 2e). Interestingly, however, the lincRNA *Peril*, which neighbors *Sox2*, is also not expressed, but showed significant enhancer activity in our MPRA data (Fig. 2f).

In total, four genes, *Morc2a*, *Lincp21*, *LincEnc1*, and *Peril*, contain high-activity peaks in their gene bodies (Log2FoldChange(Ratio) > 2). *Morc2a* has one 2019-bp high-activity region located in the middle of its gene body (Fig. 2a), while *Lincp21* contains two highly active gene body regions: one 1050-bp region near the 5′ end of the intron and one 1308-bp region near the 3′ end (Fig. 2d). *LincEnc1* contains one 1508-bp highly active region in its long central intron (Additional file 3: Figure S2F), while *Peril* contains one 2361-bp highly active gene body region located near the 3′ end of its last intron (Fig. 2f). *Lincp21* has already been established as an in vivo enhancer locus [3], and so we chose to further investigate if the presence of a high-activity MPRA peak in the gene body would also indicate in vivo DNA-based enhancer activity in another locus. We chose *Peril* for further analysis because it has one of the highest MPRA peaks, has been highlighted in previous studies [6, 21], and is of broad interest due to its association with the stem cell factor *Sox2* [23, 25].

### Enhancer candidate locus *Peril* regulates distal gene targets

**Fig. 3:**
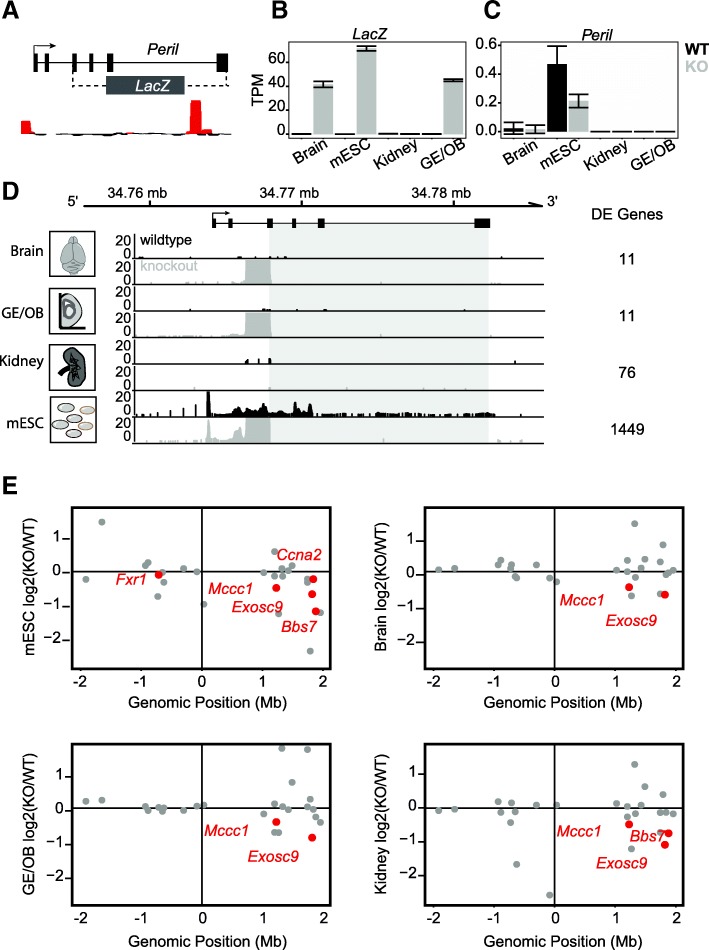
In vivo RNA sequencing validates DNA-based enhancer in *Peril* locus. **a** *Peril* locus schematic showing knockout region (dotted lines) relative to MPRA peaks (below). **b** Expression of *LacZ* and **c** *Peril* in TPMs. WT in black, KO in gray. Error bars indicate 1 standard deviation from the mean. **d** Read pileup tracks across the *Peril* locus. Gray box indicates KO region. WT in black, KO in gray. Tissue type indicated on the left. Number of dysregulated genes in WT-v-KO tissue analysis indicated on the right. **e** *Cis* plot of the 4-Mb region surrounding *Peril* (mm10) for each tissue. *Peril* locus lies at the origin. *Y*-axis represents log_2_(KO/WT), *X*-axis represents genomic position relative to *Peril* (Mb). Each dot represents a gene expressed in this tissue. Red indicates significantly differential expression between WT and KO. Genes of interest are emphasized

*Peril* stands out not only because it contains one of the strongest MPRA peaks, but also because of its genomic location. It lies 110 kb downstream of *Sox2* on murine chromosome 3 and overlaps with a super-enhancer known to regulate *Sox2* in mESC (Additional file 3: Figure S3A) [23, 25]. Using an unbiased approach to identify potential *cis*-like regulatory effects in vivo, we investigated the potential targets of the *Peril* locus. To do so, we used our *Peril* knockout mouse, in which approximately 14.5 kb of the *Peril* locus was replaced with a *LacZ* reporter (Fig. 3a, [21]). Importantly, this model removes most of the *Peril* gene body, including the high-activity MPRA region, while leaving the promoter and super-enhancer regions intact, allowing us to assess the functionality of the gene body region independently of the promoter and super-enhancer. Using this system, observation of *cis*-like regulatory events in the absence of *Peril* expression would strongly suggest that this dysregulation is due to loss of the DNA.

To determine *cis*-regulatory roles of *Peril* DNA, we performed RNA sequencing on wild-type and *Peril* knockout embryonic (E14.5) murine tissues and embryonic stem cells (Fig. 3b–d). In total, we sequenced three replicates of wild-type (WT) and knockout (KO) murine embryonic stem cells (mESC) and E14.5 kidney, and two replicates of ganglionic eminence and olfactory bulb (GE/OB). Each library was sequenced to an average depth of ~ 158 million reads, for a total of ~ 2.5 billion reads (Fig. 3b–d). We also included three *Peril* WT and KO replicates of whole brain RNA sequencing that were previously published [6].

First, we wanted to determine the expression level of both *Peril* and the *LacZ* reporter in each tissue. We found that *Peril* is transcriptionally silent in the brain, GE/OB, and kidney, but active in mESC, while *LacZ* is active in the brain, GE/OB, and mESC, but silent in the kidney (Fig. 3b, c). Next, we sought to identify any changes in gene expression between *Peril* WT and KO tissues. For each tissue, we performed differential gene expression analysis and found significant perturbations in gene expression (*p* < 0.05, DESeq alpha = 0.05, Fig. 3d, see the “Methods” section). The most striking observation was the effect on gene expression in the developing kidney, where the *Peril* region is transcriptionally silent in both the wild-type and knockout (Fig. 3d). In the kidney, we found 76 genes significantly dysregulated, 3 of which are within a 4-Mb region surrounding *Peril*. The proximity of the 3 dysregulated genes to the *Peril* locus suggests that these may be candidate targets of the *Peril* gene body enhancer (Fig. 3e).

To identify potential targets of the *Peril* locus, we queried the differentially expressed genes for every tissue, independent of the expression status of *Peril* or the *LacZ* reporter. Across all four tissues in our study, we identified only two genes that are consistently and significantly downregulated upon *Peril* deletion, and validated their expression reduction by qRT-PCR (Additional file 3: Figure S3B-C). These two genes are *Mccc1* and *Exosc9* and are located approximately 1.2 Mb and 1.8 Mb downstream of the *Peril* locus, respectively (Fig. 3e, Additional file 3: Figure S3B-C). Underscoring the potential importance of the *Peril* DNA enhancer element, *Exosc9* exhibits a prenatal lethal phenotype, consistent with an earlier observation of *Peril* knockout [21]. The physical proximity of these genes to the deleted *Peril* region suggests a potential long-range DNA-based regulatory function in the *Peril* locus.

While there are a number of mechanisms that can influence transcript level, we hypothesized that DNA elements within the *Peril* locus influence gene expression of *Mccc1* and *Exosc9*, based on our RNA-seq and MPRA data. To gain further insight, we performed a Pol II chromatin immunoprecipitation (ChIP)-qPCR experiment in *Peril* WT and KO mESC. We designed multiple sets of primers targeting the promoter regions of both candidate target genes and performed qPCR on Pol II-bound chromatin fragments. Interestingly, we found Pol II to be significantly depleted at both the *Mccc1* and *Exosc9* promoters in knockout mESC (Additional file 3: Figure S3D). These data suggest that enhancers within the *Peril* locus can regulate gene targets at a long range.

To identify which transcriptional regulators may drive the enhancer signal in the *Peril* high-activity gene body peak, we used the ChIP-atlas peak browser to identify any significant published transcription factor ChIP peaks in this region. We found evidence for binding of Duxbl1 in C2C12s, as well as multiple factors in mESC. These include Rad21 (part of the cohesin complex) and CTCF at the very beginning or immediately before the high-activity peak, as well as binding of Rxra (a retinoid-responsive factor also involved in looping), Nr4a1, and Fgfr1 within the peak (Additional file 3: Figure S3E, [29]). A noteworthy limitation of this approach is that ChIP peaks in an enhancer region are limited to which particular factors have been immunoprecipitated in relevant tissues or cells. To circumvent this limitation, we performed motif analysis of our MPRA peak region. While motif analysis is limited by a priori knowledge of specific transcription factor motifs and can be confounded by motif simplicity or redundancy, we can use this analysis to identify candidate factors within our enhancer regions. We downloaded the entire set of motifs available through the Jaspar database (vertebrate collection, [30]), and filtered these for average expression greater than or equal to 1TPM in the tissues and cells we sequenced. We then queried the sequence of the high-activity peak in the *Peril* gene body for matches to any of the expressed transcription factors on a per-sample-type basis using FIMO, a motif-finding tool publically available through the meme suite [31]. We filtered sequence matches for those with a *q* value < 0.05 and plotted the common set of expressed hits (Additional file 3: Figure S3F). From this analysis, we find factors that may bind and drive the enhancer activity in this region: Rarg (another retinoid-responsive factor), Myc, and Foxk1. Taken together, these combined analyses identified a number of candidate factors that could influence the enhancer activity identified in the *Peril* locus, and pose multiple interesting directions for future study into exactly how this gene body enhancer regulates target gene expression.

### Chromosome conformation capture reveals altered interactions with regulatory targets in *Peril* knockout mESCs

**Fig. 4:**
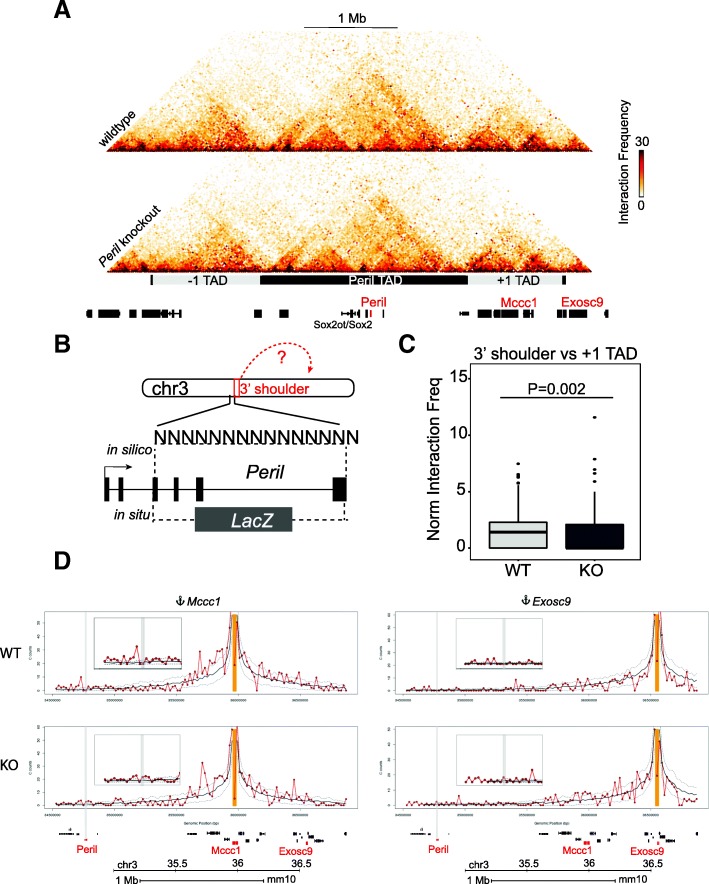
Hi-C analysis of WT-v-*Peril* KO mESC. **a** Chromatin contact map surrounding the *Peril* locus at 20-kb resolution using custom mm10 genome build in wild-type (top) and knockout (bottom). Genes indicated below, with genes of interest indicated in red. TAD borders indicated in black; − 1 and + 1 TADs demarcated by gray box while *Peril*-containing TAD is demarcated by a black box. **b** Mapping strategy of the Hi-C data to a custom mm10 mouse genome, where the deleted *Peril* region (~ 14.5 kb) is replaced by Ns and this sequence or the *LacZ* (~ 3.5 kb) are compiled as separate chromosomes. 3′ shoulder region (not affected by deletion, but used as a bait region common to both WT and KO conditions) indicated in red. **c** Digital 4C summary of normalized interactions between the 3′ shoulder region (chr3:34784809–34800385) and the + 1 TAD in either WT or KO. **d** Digital 4C plots of the interaction anchored on either *Mccc1* or *Exosc9* in WT and *Peril* KO Hi-C samples. The red line indicates the distance-normalized average interaction frequency, whereas the dashed lines represent the ± 1 standard deviation. The 20-kb bins that are above the + 1 standard deviation line are considered as enriched interactions. The red arrows indicate the alterations of long-range interactions of *Mccc1* and *Exosc9* with the *Peril* locus between WT and KO samples

To assess how *Peril* ablation affects regulatory interactions and higher-order chromatin conformation changes, we performed high-throughput chromosome conformation capture (Hi-C) on WT and *Peril* KO mESC (Fig. 4a, Additional file 3: Figure S4A), [32, 33]. Briefly, two replicates of WT and KO mESC cells were sequenced to an average depth of approximately 155 million combined reads and analyzed using the HiC-Pro pipeline [34]. Both sets of replicates showed high reproducibility (Additional file 3: Figure S4B-C), and reads across the *Peril* locus confirmed WT and KO genotypes (Additional file 3: Figure S4D).

The TAD structure surrounding the *Peril* locus consists of a series of small sub-TADs contained within a 2-Mb TAD, while the two adjacent TADs (− 1 TAD and + 1 TAD) are smaller in size: ~ 900 kb and ~ 1 Mb, respectively (Fig. 4a). The 2-Mb *Peril*-containing TAD contains only a few genes and one centrally located sub-TAD, only ~ 150 kb in size, which contains *Peril*, the super-enhancer, and *Sox2*. Interestingly, we found that *Peril* deletion does not lead to gross chromatin conformation changes on chromosome 3 (Fig. 4a), nor does it disrupt the ~ 150-kb sub-TAD containing *Peril* and *Sox2*. Thus, despite the association of *Peril* with *Sox2* within this sub-TAD, removal of DNA elements contained in the deleted region do not influence local architecture around the *Sox2* locus. This is consistent with our finding that there is no significant expression effect on *Sox2* in any of the *Peril* knockout tissues and cell lines tested (Fig. 3). Thus, the majority of the *Peril* gene body is dispensable for *Sox2* regulation.

To identify which regions on chromosome 3 interact specifically with the deleted region, we generated a custom mm10 genome build which replaces the deleted region with spacer nucleotides (Ns) and contains two separate chromosomes: one containing the deleted region sequence and one containing the *LacZ* sequence (Fig. 4b). We sought to perform a digital 4C-like analysis anchoring on the “deleted region” chromosome or “LacZ” chromosome in WT or KO samples, respectively, but were limited by the lack of restriction enzyme sites in the LacZ sequence (see the “Methods” section). Unable to directly compare the deleted region to the LacZ bait, we instead compared interactions from the 3′ neighboring 20-kb bin and found a significant decrease in interactions between WT and KO with the + 1 TAD (see the “Methods” section, Fig. 4c, Additional file 3: Figure S4E). This indicates that, in the KO, these interactions with the neighboring regions are altered (Fig. 4c, Additional file 3: Figure S4E).

It has previously been shown that expression of genes within a TAD are correlated and that while TAD boundaries tend to insulate interactions between neighboring TADs, inter-TAD interactions do occur [35–40]. It is thus quite interesting to note that both *Mccc1* and *Exosc9* are located within the + 1 TAD relative to *Peril*. To gain insight into potential long-range regulatory effects of *Peril* deletion, we generated digital 4C-like plots anchored on either *Mccc1* or *Exosc9*. Indeed, upon loss of *Peril*, we observe an alteration of contacts between these loci and the region just upstream of the *Peril* deletion (Fig. 4d). Specifically, *Mccc1* appears to lose interactions with this region in KO cells, while the *Exosc9* region appears to demonstrate a subtle gain of interactions. Thus, in our RNA-seq dataset, *Mccc1* and *Exosc9* are transcriptionally downregulated in all tissues and cells, and in our Hi-C dataset, these targets appear to show altered contacts with *Peril*. This, in combination with the decrease in Pol II binding at the target promoters in *Peril* knockout mESC, suggests that *Mccc1* and *Exosc9* are the targets of *Peril* DNA enhancers.

Together, our results indicate that the *cis*-regulatory elements at the *Peril* locus show altered interactions with the + 1 TAD, which harbors the two consistently dysregulated genes, *Mccc1* and *Exosc9.* These results provide evidence for enhancer elements encoded in the *Peril* locus that play a role in the regulation of *Mccc1* and *Exosc9* expression. Ultimately, these data highlight the importance of considering DNA regulatory roles in examining any given genic locus.

## Discussion

Recently, it has been shown that lincRNA loci influence the expression of their neighboring protein-coding genes [7, 22, 41–43]. However, the mechanisms of this regulation are largely uncharacterized. Here, we selected candidate lincRNAs based on their potential *cis*-regulatory function and directly tested the enhancer capacity of their loci at 50-bp resolution using MPRA technology. Since we sought to test whether or not lincRNA loci would contain more enhancers or more highly active enhancers than protein-coding gene loci, we quantitatively mapped the enhancer capacity of a set of candidate *cis*-functional lincRNAs and their closest protein-coding neighbors.

Recent studies have suggested that promoters may play a larger role in *cis*-regulatory dynamics than previously appreciated [8–12]. Specifically, promoters are capable of exerting *cis*-regulatory function on neighboring genes in addition to promoting expression of the immediately adjacent sequence. Consistent with these studies, we found that indeed, the most enhancer activity within a locus, whether lincRNA or mRNA, occurs within the region 1 kb upstream of a TSS. However, we also identified DNA regulatory elements with varying activity levels within gene bodies in both mRNAs and lincRNAs. By combining a reporter knockout mouse model, in which the gene body but not promoter has been removed, with an MPRA (employed as a high-resolution enhancer mapping technique), we are able to identify novel enhancers as well as their candidate target genes. Together, these results underscore the importance of gene body DNA regulatory elements contained in both lincRNA and mRNA loci.

Resolving the targets of these DNA regulatory elements is nontrivial. To untangle modalities of regulation at lincRNA loci and to identify potential gene targets, we used the *Peril* locus as a model. The *Peril* locus overlaps with a *Sox2* super-enhancer and forms a tightly associated sub-TAD with the *Sox2* locus (Fig. 4b, [23, 25]). It also generates a spliced lincRNA and, by *LacZ* reporter expression, appears to be active in tissues that express *Sox2* [6, 21, 44]. These findings all suggest a connection between *Peril* and *Sox2*. However, using unbiased RNA-seq approaches in vivo, we found no significant evidence for *Peril* regulation of *Sox2*. Instead, we discovered consistent and significant downregulation of two genes located within the neighboring TAD: *Exosc9* and *Mccc1*. This method of using RNA sequencing across a variety of tissues to identify consistently misregulated genes, particularly in tissues where the lincRNA is not transcribed, has been used previously to identify the targets of DNA-based enhancer elements [3].

Comparison between wild-type and *Peril* knockout Hi-C samples reveals alterations in interactions between the *Peril* region and the TAD containing the candidate target genes. It has been previously established that transcriptional co-regulation of genes is strongly associated with TAD formation [35, 40] and that regulatory modules can affect TAD-wide gene expression [37, 39]. The observation that *Peril* shows altered interaction frequency with target genes at the + 1 TAD, (Figs. 3e and 4c, d, Additional file 3: Figure S4E) suggests a possible functional role for *Peril* in the transcriptional regulation of these genes by long-range chromatin interactions. In part, this could occur by DNA elements within the *Peril* locus influencing Pol II occupancy at the *Mccc1* and *Exosc9* promoters, which is supported by our ChIP-qPCR data (Additional file 3: Figure S3D). Yet, the exact mechanisms of the establishment and maintenance of these long-range interactions remain to be determined. Some insight is provided by our intersection of publicly available ChIP-seq data with the *Peril* enhancer region identified by MPRA, in which we have identified potential binding of important genome organizer factors such as Rad21 and Ctcf, as well as Rxra. All of these factors have been significantly implicated in chromatin looping and enhancer function [29, 45, 46].

Consistent with our previous report of perinatal lethality among *Peril* knockout mice [21], loss of *Mccc1* and *Exosc9* are documented to have deleterious effects in human and mouse, respectively, including prenatal lethality [47–50]. We find that both *Mccc1* and *Exosc9* are consistently and specifically downregulated as a consequence of *Peril* ablation. Consistent with *Peril* targeting *Mccc1* and *Exosc9*, we observed fewer physical interactions exist between the *Peril* locus and the TAD containing these genes in knockout mESC. These data point to a potential in vivo DNA-based mechanism for *Peril*: function through control of distally located, developmentally important genes. Although we can rule out the role of the *Peril* RNA in regulating some target genes (e.g., *Mccc1* and *Exosc9*), we cannot exclude an additional role for the RNA *in trans*. Consistent with this possibility, we observed numerous gene expression changes in the *Peril* knockout mESC line which could be due to loss of the RNA product, though this effect could also be due to a cell type-specific effect of reduced *Mccc1* or *Exosc9* transcript levels.

Beyond *Peril*, our study further highlights the DNA regulatory potential contained in promoters and gene bodies regardless of coding classification (i.e., lincRNA or mRNA). We found that both lincRNAs and mRNAs contain *cis*-regulatory potential in their promoters, as described previously [8–12, 15]. Furthermore, we demonstrate that in both gene types, regulatory potential is additionally contained in gene bodies, though we found that high-activity regions occurred in more of the lincRNA (*Lincp21*, *LincEnc1*, and *Peril*) than mRNA (*Morc2a*) gene bodies we queried.

The presence of clear enhancer activity in these loci has important implications for our current understanding and interpretation of lincRNA knockout models, which have been the focus of intense debate [5, 22]. Specifically, these results highlight the potential for a given locus to generate a spliced gene product and also contain functional DNA elements. This work and others [3, 4] highlight that the functions of these two molecular species should not be conflated, but rather independently assessed. It should also be noted that mRNA gene bodies have been known to harbor enhancers as well, as established by numerous ChIP-seq experiments, or conservation mapping [13, 51]. For example, *Dync1l1* (an mRNA locus) harbors exonic enhancers which regulate nearby genes *Dlx5/6*, and removal of these regulatory sequences can lead to disease [18]. Similarly, an intronic sequence in *Lmbr1* acts over 1 Mb to regulate *Shh* expression in the developing limb [51]. Currently, hundreds of knockout strains and ESC lines targeting mRNA loci have been generated using whole gene ablation techniques (for example, by the immense efforts of the *K*nock*o*ut *M*ouse *P*roject (KOMP) and the *I*nternational *M*ouse *P*henotyping *C*onsortium (IMPC)) [50, 52–54]. Some of these strains use the same whole locus gene targeting technique as our lincRNA mouse models, which means that the same complications will arise in resolving the functional molecular species at these mRNA loci. Here, we provide a logical framework for follow-up studies in these models to identify DNA elements and their regulatory targets.

## Conclusions

Here, we conclude, by combining MPRA, in vivo deletion/reporter models, and Hi-C, that both mRNAs and lincRNAs can contain DNA regulatory elements in their gene bodies. Further investigation of one of these DNA elements in vivo revealed a surprising finding: that *Peril* regulates two distally located genes (*Mccc1* and *Exosc9*) and not its neighbor *Sox2*. Despite close proximity of the *Peril* locus with *Sox2*, we find that neither the DNA nor the RNA in the *Peril* locus affect *Sox2* expression. Moreover, we find that ablation of *Peril* does not affect the local chromatin architecture, but does affect long-range interactions with *Mccc1* and *Exosc9* in the neighboring TAD. Together, our combined approach is able to identify DNA regulatory elements in lincRNAs and their candidate regulatory targets in vivo.

## Methods

### Massively parallel reporter assay

The MRPA pool was designed as previously described [3, 55], with the following important alterations. (1) The full gene body of each locus and 1000 bp upstream (the promoter region) were included in the design. (2) Five micrograms of GFP+ plasmid was used to transfect C2C12 cells in triplicate wells of six-well dishes using lipofectamine 2000 (Thermo Fisher 11668027) and low-serum, antibiotic-free media. RNA was harvested using TRIzol, and libraries were generated as described previously [3, 55]. Five replicate samples were generated for RNA samples (taken from biological replicates at two different passages), as for DNA samples (generated on two separate days). Libraries were generated via PCR with indexed primers. For RNA libraries, 1 μg RNA input was used, while for DNA libraries, 1 ng of the MPRA vector was used. Samples were sequenced on a Hiseq 2500 at Harvard University’s Bauer Sequencing Core.

### C2C12 culture

C2C12 cells were acquired from ATCC (CRL-1772) and cultured in DMEM supplemented with FBS and antibiotics.

### MPRA analysis overview

Analysis was performed largely as described previously [3] with three important differences in the calculation of the activity ratio between RNA and DNA replicates. (1) After normalizing for sequencing depth, each sample was median-normalized. (2) The activity ratio for each barcode was determined by randomly selecting one RNA replicate and one DNA replicate and calculating the ratio normed_RNA/normed_DNA. This process was repeated 1000 times, and the resulting median was reported as the final signal ratio for that barcode. (3) Calculation of significant regions was performed by shuffling sample labels rather than barcode labels. In other words, we repeated step 2 without labeling which samples were RNA or DNA to generate a null distribution against which to compare true signal in any given region. *p* values were corrected using the Benjamini-Hochberg method with an FDR of 1%. All analysis was performed using the aid of in-house python and R scripts, with aid from biopython and R’s bioconductor packages. Oligo annotation is available as Additional file 1, TSS annotation is available as Additional file 2, and all code is available as Additional file 4. Additionally, code is available under the MIT License on github (https://github.com/agroff11/PerilPaperAnalysis) and on zenodo (10.5281/zenodo.1477113) [56].

### Mice

Mice were housed under pathogen-free conditions at Harvard University. A description of the mice used in this experiment is available as Additional file 5. The *Peril* knockout strain was originally generated by Regeneron Pharmaceuticals [21, 57].

### Derivation and culture of mESC

Wild-type and *Peril* knockout murine ESCs were generated by the Harvard University Genome Modification Facility from D3.5 embryos. After receiving clones from the core, mESC colonies were transitioned to 2i on feeder MEFs and cultured for six passages. Three individual wells (derived from the same original clone) were collected as biological replicates.

### Tissue collection

Three biological replicates of the kidney and two biological replicates of microdissected ganglionic eminence and olfactory bulb (GE/OB) were harvested from E14.5 progeny of heterozygous matings. Tissues were pulverized in TRIzol and snap frozen.

### RNA library generation and sequencing

The Illumina TruSeq kit was used to create polyA^+^ libraries from 250 ng total RNA for each tissue replicate and 500 ng total RNA for each mESC replicate. Samples were sequenced on a Hiseq 2500 at Harvard University’s Bauer Sequencing Core.

### Sequence alignment and analysis

Reads were aligned to mm10 and quantified using rsem with nonstandard parameters --paired-end --bowtie2 --append-names and -p 8 [58]. Read counts from this quantification were used in downstream differential expression analysis using DESeq (alpha = 0.05 for all comparisons, [59]). Reads in the knocked out region of *Peril* were assessed visually to confirm knockout status in combination with expression status of *LacZ*. All RNAseq figures were generated in R with the help of packages available on bioconductor. Code is available as Additional file 6 and is available under the MIT license on github (https://github.com/agroff11/PerilPaperAnalysis) and on zenodo (DOI: 10.5281/zenodo.1477113) [56].

### qRT-PCR

cDNA was generated using SuperScriptIII (Thermo Fisher), and qRT-PCR analyses were performed using an ABI7900HT Fast Real-Time PCR system (applied biosystems). Data was analyzed using qPCR-miner [60], and the ddCT method was used to calculate relative cDNA. Samples were normalized to L32 control. Primers used are as follows: Mccc1 (L) gggtgtgcagtcggtggctg, Mccc1 (R) gcctggatggatggcctgtgc, Exosc9 (L) aagagcgtgatcccgtgcca, Exosc9 (R) tggcaatcaccagcaagccatcc, L32 (L): aacccagaggcattgacaac, L32 (R): attgtggaccaggaacttgc.

### Transcription factor binding analysis

We downloaded the entire set of motifs available through JASPAR (the JASPAR vertebrate collection, [30]) and filtered these for an average expression greater than or equal to 1TPM in the tissues and cells used in this paper. We then queried the sequence of the high-activity peak in the *Peril* gene body for matches to any of the expressed transcription factors on a per-sample-type basis using FIMO, a motif-finding tool publically available through the meme suite [31]. We filtered sequence matches for those with a *q* value < 0.05 and plotted the binding profiles of the common set of expressed hits. Additionally, we assessed publically available TF binding via the ChIP-atlas [61].

### Chromatin immunoprecipitation (ChIP)

ChIP experiments were performed using the iDeal ChIP-qPCR kit (Diagenode, C01010180), and experiments were carried out according to the manufacture protocol. Briefly, approximately 16 million wild-type or 16 million *Peril* knockout mESC were crosslinked for 10 min in 1% formaldehyde. Chromatin was sheared with the following parameters: 15 min of 30 s “on” and 30 s “off” using the Bioruptor (Diagenode). ChIP experiments were performed using 2 μL of Pol II monoclonal antibody (Diagenode, C15100055) per ChIP, and as negative control, 1 μg of IgG was used in a separate ChIP. For downstream enrichment calculation, 1% of the input chromatin was retained. The occupancy of Pol II at the promoter of Mccc1 and Exosc9 was measured by real-time qPCR using two primer sets targeting different regions of the promoters. qPCR was performed using FastStart Universal SYBR Green Master (Rox) (ABI, 4913914001), and reactions were run on ViiA7 Real-Time PCR System (ABI). Primer sets used in the experiment targeted Pol II-enriched regions near the target gene promoters as well as control regions: F1_Mccc1: CAAGTAGGCGTCTCCGCAA; R1_Mccc1: GGTCCAATCAGCCAATCGGTA; F2_Mccc1: ATTCTTGAAAGCGTCCCTCCC; R2_Mccc1: CTTGCGGAGACGCCTACTTG; F3_Exosc9: ACATTGTCAGTTCGGCCTGT; R3_Exosc9: AATACTGCCCTGAGGCTTGG; F4_Exosc9: GTGTGGACTCTCCCCATTCC; R4_Exosc9: TCCCTGCCACTGAATACTGC; F_Pol2_positive_region: CTGGCACTGCACAAGAAG; R_Pol2_positive_region: GGGTTCCTATAAATACGG; F_Pol2_negative_region: CTGGCCTCCATACACACATA; R_Pol2_negative_region: AGTCAGCAGGATCCACACTT. Enrichment was calculated by the percent input method.

### Generation and analysis of Hi-C libraries

In situ Hi-C libraries were generated using the *HindIII* restriction enzyme [32, 33]. Briefly, ~ 25 million cells were crosslinked with 1% formaldehyde for 10 min at room temperature. Then, the chromatin was extracted, digested with *HindIII*, end-labeled with biotin-14-dCTP, followed by an in situ ligation procedure. After DNA extraction, biotin was removed from unligated ends, and the sample was sheared by using a Covaris S220 instrument (100–500 bp range). After A-tailing, biotin pull-down and adapter ligation, paired-end sequencing was performed on a HiSeq instrument. Each Hi-C library was generated in two biological replicates, and each replicate was sequenced to a depth of ~ 80 million reads. Hi-C mapping, filtering, iterative correction, and binning were performed with the HiC-Pro software v2.8 [34]. We used multiple genome builds in our analyses: for general quality control and structural analyses, we used both mm9 and mm10, and for the digital 4C analyses, we used a custom mm10 genome which replaced the deleted *Peril* region with Ns and then included this sequence as well as the LacZ reporter sequence as two separate chromosomes.

There was a high correlation among the Hi-C biological replicates, and so we pooled all biological replicates for each condition and analyzed them as a single Hi-C dataset. This pooled dataset was used for all the analyses. The TAD analysis was performed with the insulation method, as previously described [62], by using the *cworld* package (https://github.com/dekkerlab/cworld-dekker) [63] “matrix2insulation.pl” script with the following options: –is 480000 –ids 320000 –im iqrMean –nt 0 –ss 160000 –yb 1.5 –bmoe 0 –bg. The digital 4C plots were generated with the *cworld* package “matrix2anchorplot.pl” script and with the HiC-Pro “make_viewpoints.py” script by using either the *Mccc1* or the *Exosc9* loci as anchor points. To do this, we calculated the Hi-C interaction frequency between the Mccc1/Exosc9-containing bins and the neighboring 20-kb bins. WT interactions were mapped to the mm10 reference genome while KO interactions were mapped to our custom mm10 genome build. For each distance, the ± 1 standard deviation of all the Hi-C interaction frequencies of that distance (dashed lines in the figure) were also calculated. The interactions that were above the + 1 standard deviation line (i.e., positive *z*-score) were considered as enriched interactions. To investigate the interactions between the *Peril* region and the neighboring TAD, we first investigated the *HindIII* restriction sites in the deleted region or LacZ “chromosomes.” We found that the “deleted region chromosome” contains three cut sites while the “LacZ chromosome” contains none. We thus selected the 3′ shoulder region (encompassing the 6 downstream *HindIII* sites, ~ 15 kb, chr3:34784809–34800385). This region is shared between WT and KO and is not affected by the deletion. While not specific to the deleted sequence, it can be used as a proxy for alterations in interactions between WT and KO in the deleted region. To generate digital 4C-like bedgraphs anchored on this shoulder region, we plotted either raw reads originating in this region and ending on chromosome 3, or a boxplot of all normalized reads originating from this region and ending in the + 1 TAD. The boxplot was generated using the custom genome for both WT and KO samples.

## Additional files

Additional file 1: MPRA oligo annotation - File containing annotation information used to associate unique barcode tags to synthesized genomic elements in the MPRA experiment. (TXT 7035 kb)

Additional file 2: TSS annotation – File containing gencode TSS information for loci used in this study. (TXT 891 bytes)

Additional file 3: Supplemental Figures - File contains Figures S1-S4 and their legends. (PDF 5159 kb)

Additional file 4: MPRA analysis code - R markdown file containing all code used in the MPRA analysis. (RMD 38 kb)

Additional file 5: Mouse master sheet - File containing description of mice used in this study, and information about generation of RNA sequencing libraries. (XLSX 52 kb)

Additional file 6: RNA seq analysis code - R markdown file containing all code used in the RNA-sequencing analysis. (RMD 15 kb)
