# Supplementary material for: Enhancers in the Peril lincRNA locus regulate distant but not local genes
Source: Genome Biol. 2018 Dec 11;19:219. doi: 10.1186/s13059-018-1589-8 (PMC6290506; doi:10.1186/s13059-018-1589-8)
Supplement: Supplementary file 3 — Supplemental Figures - File contains Figures S1-S4 and their legends. (PDF 5159 kb) [file 13059_2018_1589_MOESM3_ESM.pdf]

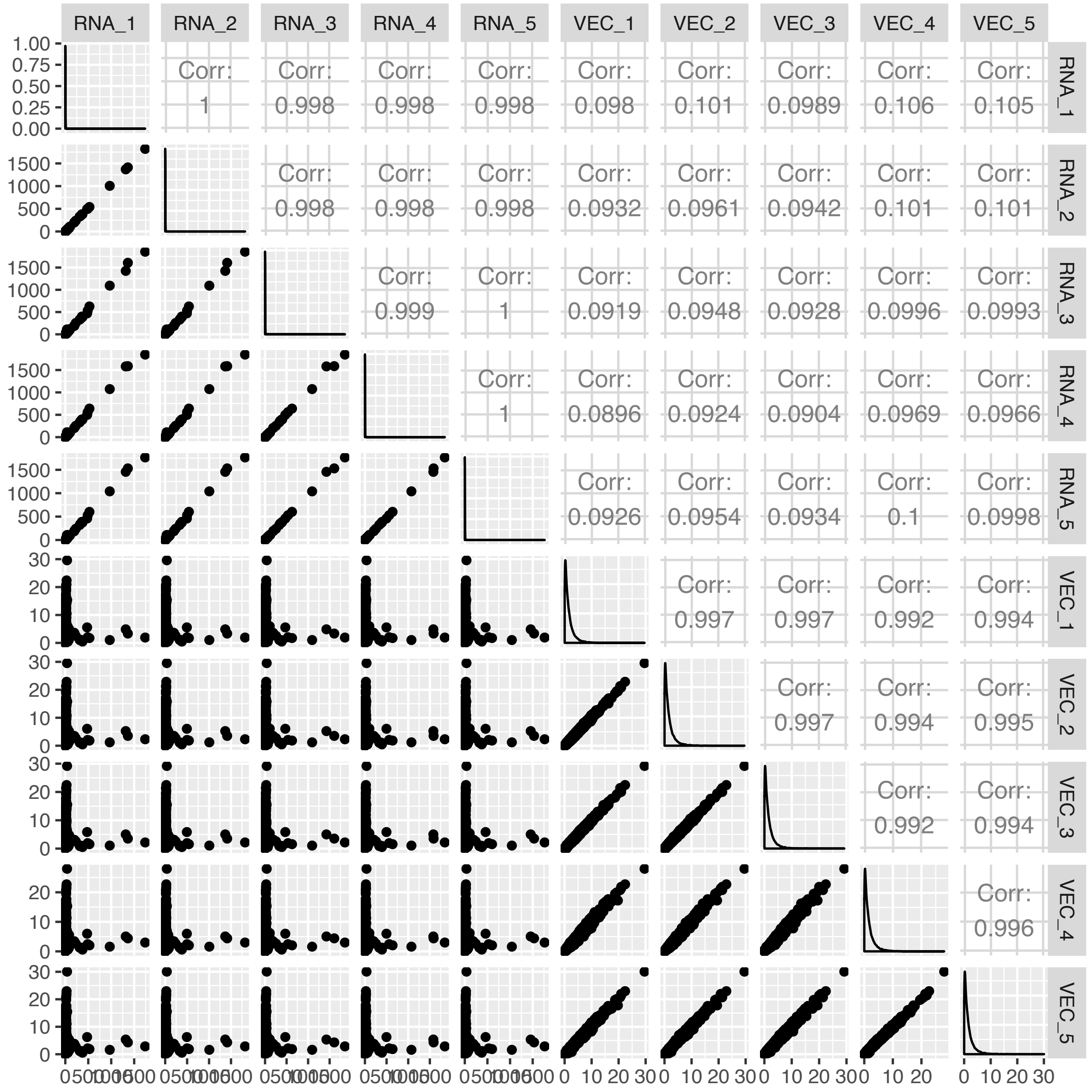

Figure S1: Supplement to MPRA quality control - pairwise scatterplots of all MPRA RNA and DNA samples.

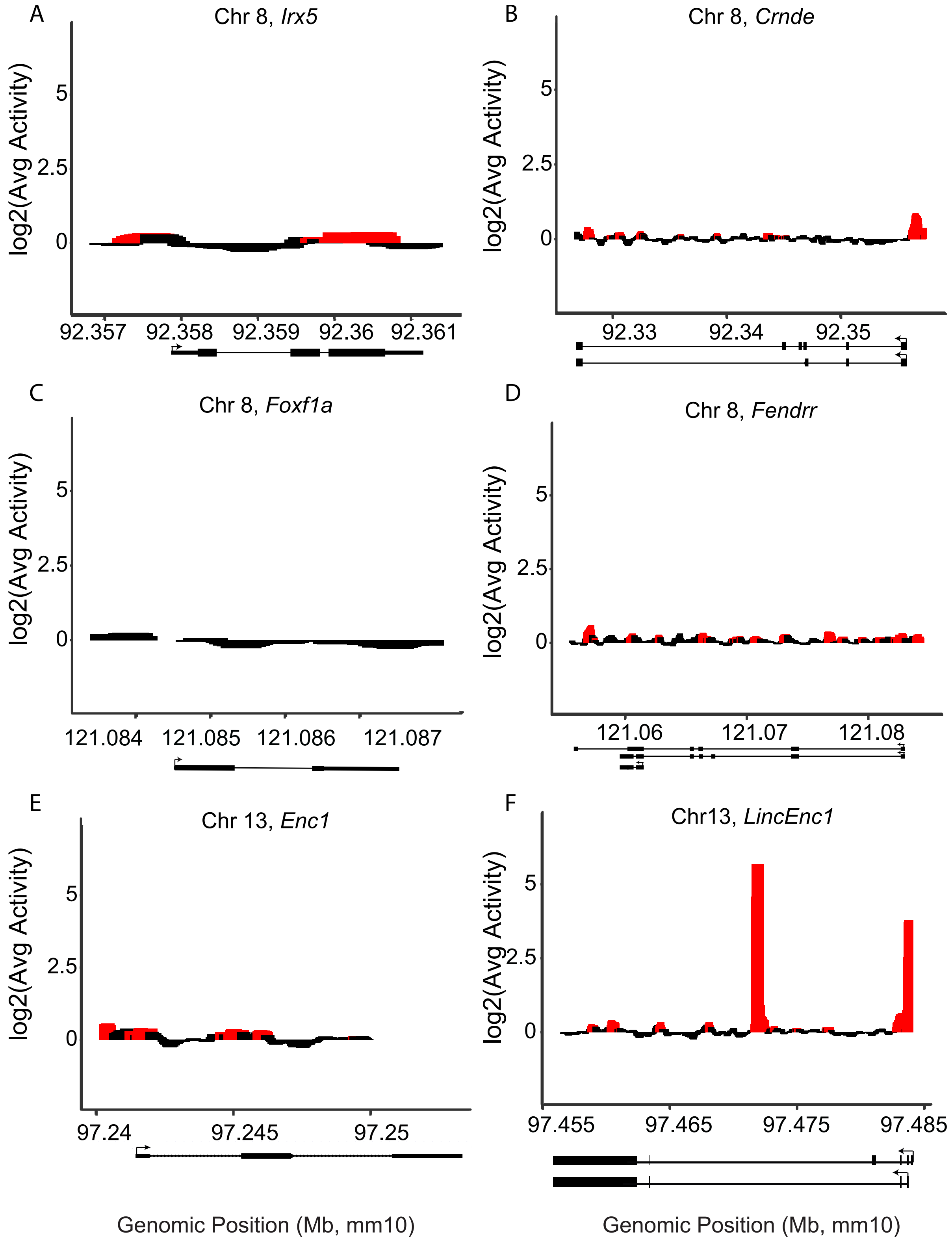
A
Chr 8, *Irx5*
B
Chr 8, *Crnde*
C
Chr 8, *Foxf1a*
D
Chr 8, *Fendrr*
E
Chr 13, *Enc1*
F
Chr13, *LincEnc1*
log2(Avg Activity)
5
2.5
0
92.357
92.358
92.359
92.36
92.361
92.33
92.34
92.35
121.084
121.085
121.086
121.087
121.06
121.07
121.08
97.24
97.245
97.25
97.455
97.465
97.475
97.485
Genomic Position (Mb, mm10)
Genomic Position (Mb, mm10)

Figure S2: Supplement to MPRA enhancer activity results - smoothed region plots for (A) *Irx5*, (B) *Crnde*, (C) *Foxf1a*, (D) *Fendrr*, (E) *Enc1*, and (F) *LincEnc1*. Red indicates significantly activated regions (see methods). Genomic position (in Mb, mm10) across the X axis, with gene structures indicated below. Y axis represents $\log_2$(activity).

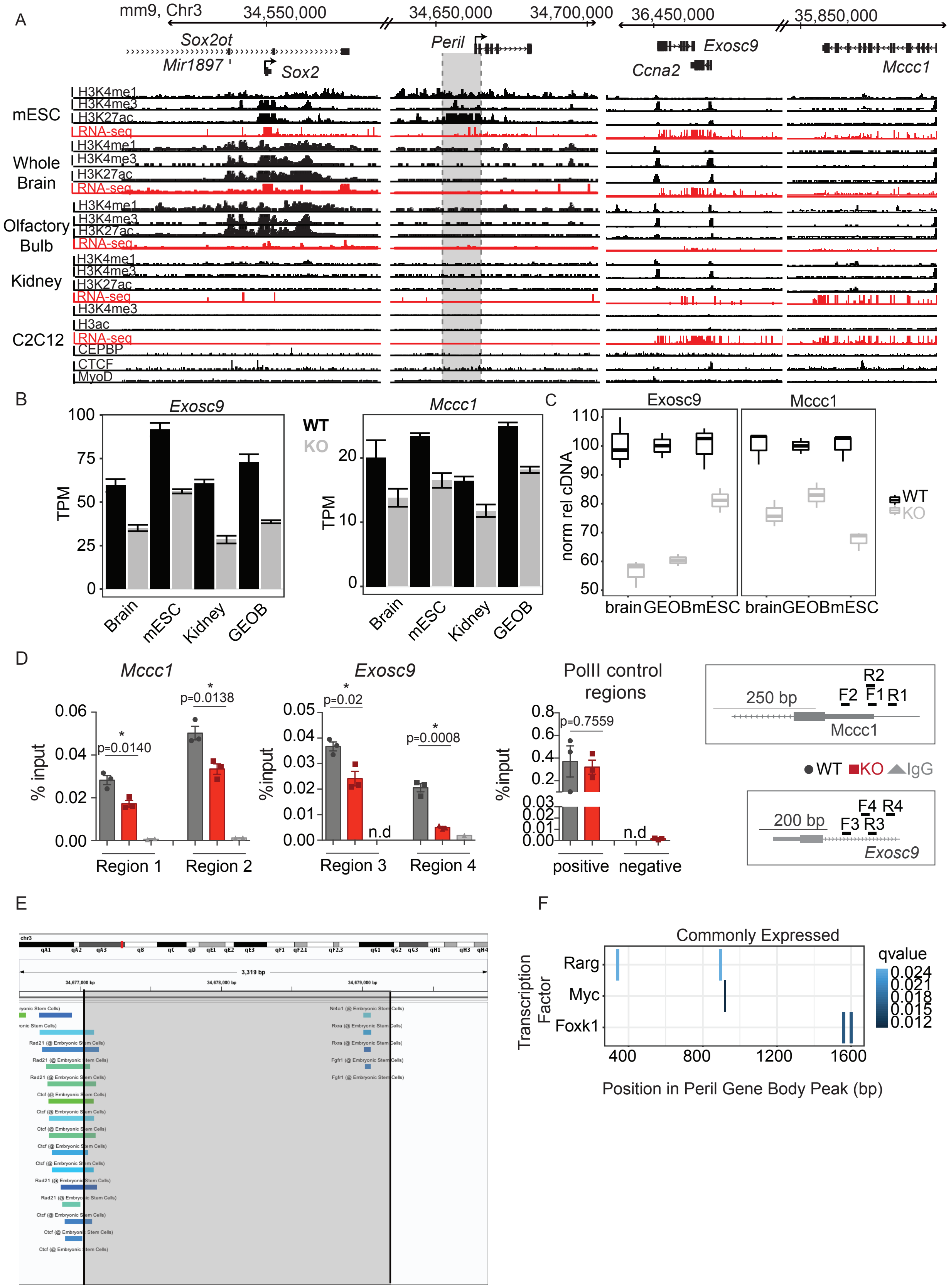

A
mm9, Chr3
34,550,000
34,650,000
34,700,000
36,450,000
35,850,000
Sox2ot
Mir1897
Sox2
Peril
Exosc9
Ccna2
Mccc1
mESC
Whole Brain
Olfactory Bulb
Kidney
C2C12
H3K4me1
H3K4me3
H3K27ac
RNA-seq
H3ac
CEPBP
CTCF
MyoD
B
Exosc9
Mccc1
WT
KO
TPM
100
75
50
25
0
20
10
Brain
mESC
Kidney
GEOB
C
norm rel cDNA
110
100
90
80
70
60
50
brain GEOB mESC brain GEOB mESC
D
Mccc1
Exosc9
PolII control regions
% input
p=0.0140
p=0.0138
p=0.02
p=0.0008
p=0.7559
n.d
Region 1
Region 2
Region 3
Region 4
positive
negative
250 bp
F2 F1 R1
R2
200 bp
F4 R4
F3 R3
WT KO IgG
E
chr3
3,319 bp
F
Commonly Expressed
Transcription Factor
Rarg
Myc
Foxk1
400
800
1200
1600
Position in Peril Gene Body Peak (bp)
qvalue
0.024
0.021
0.018
0.015
0.012

Figure S3: Supplement to *Peril in vivo* RNAseq analysis. (A) Publicly available ChIP-seq and RNAseq tracks for five diverse murine tissues or cells (indicated in left margin), showing the *Peril* and *Sox2* loci, as well as *Mccc1* and *Exosc9* on chromosome 3 (UCSC genome browser, mm9). Grayed region indicates the super enhancer. All track Y-axes scaled 0-15. (B) Expression in TPMs for two target genes *Exosc9* and *Mccc1*. Wild-type samples indicated in black and knockout in gray. (C) qRT-PCR validation of expression values for *Exosc9* and *Mccc1* normalized to L32. Y-axis indicates normalized relative cDNA (relative to WT, normalized to L32). (D) Assessment of Pol-2 binding at promoters of *Mccc1* and *Exosc9*. Two different regions at the *Mccc1* or *Exosc9* promoters in WT (dark gray) and *Peril* KO (red) mESC (n=3). Control IgG ChIP from WT and *Peril* KO shown together (light gray). Control IgG ChIP from WT and *Peril* KO shown together (light gray). Pol-2 ChIP enrichment shown as 1 percent (%) input. Error bars indicate the standard error of the mean (s.e.m). Significance determined by an unpaired two-tailed t-test. 'n.d' indicates not determined. Primer locations depicted in schematic on the right. (E) ChIP-Atlas screenshot of the *Peril* gene body high activity peak region (shaded in gray), displaying all transcription factor binding sites in publically available mESC data using the most permissive cutoff (-10Log10(MACS2 Q-value) > 50). (F) FIMO analysis of the *Peril* gene body peak sequence using transcription factors expressed >1TPM in all tissues/cells used in this paper (C2C12 and mESC cells, E14.5 whole brain, GE/OB, and kidney). X-axis represents position within the peak (bp) and color indicates FIMO analysis q-value. Width is representative of the binding site width.

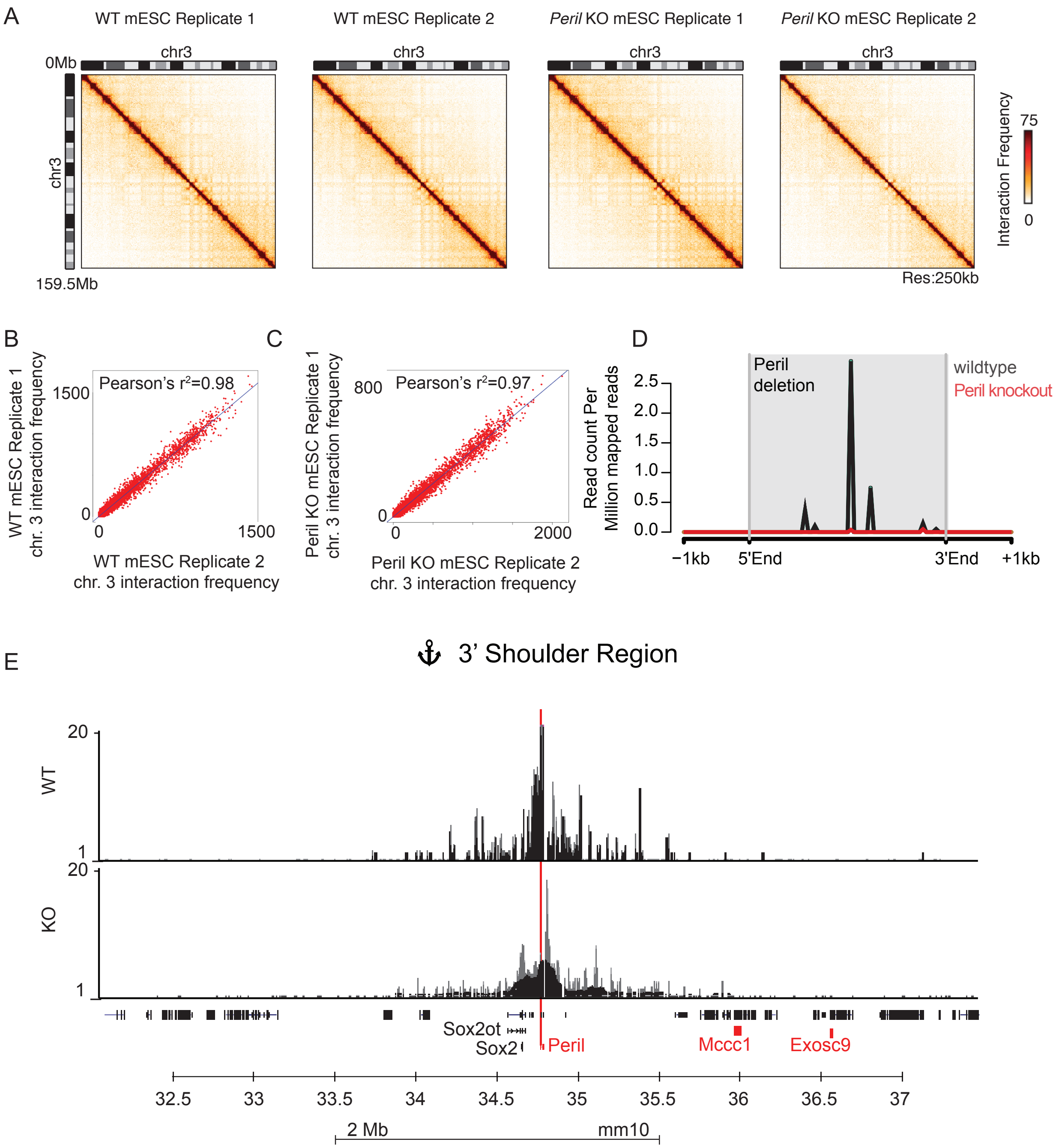

A
WT mESC Replicate 1
WT mESC Replicate 2
Peril KO mESC Replicate 1
Peril KO mESC Replicate 2
chr3
0Mb
159.5Mb
Interaction Frequency
75
0
Res:250kb
B
WT mESC Replicate 1 chr. 3 interaction frequency
Pearson's r²=0.98
1500
0
WT mESC Replicate 2 chr. 3 interaction frequency
C
Peril KO mESC Replicate 1 chr. 3 interaction frequency
Pearson's r²=0.97
800
2000
Peril KO mESC Replicate 2 chr. 3 interaction frequency
D
Read count Per Million mapped reads
Peril deletion
wildtype
Peril knockout
2.5
2.0
1.5
1.0
0.5
0.0
−1kb
5'End
3'End
+1kb
E
3' Shoulder Region
WT
KO
20
1
Sox2ot
Sox2
Peril
Mccc1
Exosc9
32.5
33
33.5
34
34.5
35
35.5
36
36.5
37
2 Mb
mm10

Figure S4: Supplement to WT-v-Peril KO Hi-C Data (A) Chromatin contact maps for all of chromosome 3 in each WT and *Peril* KO replicate at 250kb resolution (mm9 reference genome). (B) Scatter plot of reads from HiC WT replicate 1 vs WT replicate 2 and (C) KO replicate 1 vs KO replicate 2. (D) Read pileups over *Peril* KO region in the HiC data +/- 1Kb. WT in black, KO in red, deleted region indicated by gray box. Y axis indicates reads per million mapped reads. (E) 4C-like bedgraphs depicting raw reads mapping to chromosome 3, where the anchor is the 3' shoulder region in the WT or *Peril* KO Hi-C samples. Genes of interest marked in red, and deletion region indicated with a red line.
